# Supplementary material for: A Probiotic Lactobacillus gasseri Alleviates Escherichia coli-Induced Cognitive Impairment and Depression in Mice by Regulating IL-1β Expression and Gut Microbiota
Source: Nutrients. 2020 Nov 10;12(11):3441. doi: 10.3390/nu12113441 (PMC7697804; doi:10.3390/nu12113441)
Supplement: Supplementary file 1 [file nutrients-12-03441-s001.pdf]

[Supplementary Material]

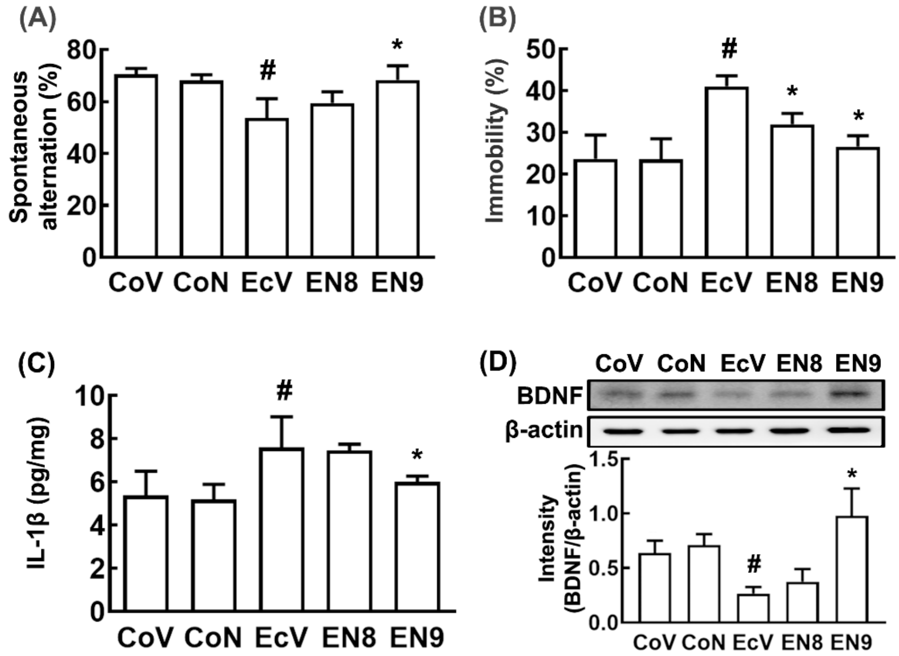

Figure S1. NK109 alleviated K1-induced cognitive impairment and depression in mice. (A) Effect on the memory impairment in Y-maze task. (B) Effect on the depressive behaviors in the TST (C). Effect on the IL-1β level in the hippocampus. (D) Effect on BDNF expression in the hippocampus. Mice of EcV, EN8, and EN9 groups were exposed to K1 (1×10^9 CFU/mouse/day) daily for 5 days and thereafter treated with test agents (EcV, vehicle; EN8, 1×10^8 CFU/mouse/day of NK109; EN9, 1×10^9 CFU/mouse/day of NK109) for 5 days. Control group (CoV) was treated with 1% maltose instead of K1 and NK109 and CoN group was treated with 1% maltose instead of K1 for 5 days and thereafter NK109 (1×10^9 CFU/mouse/day of NK109) was orally gavaged for t days. Data values were indicated as mean ± SD (n = 6). Means with same letters are not significantly different ($p < 0.05$).

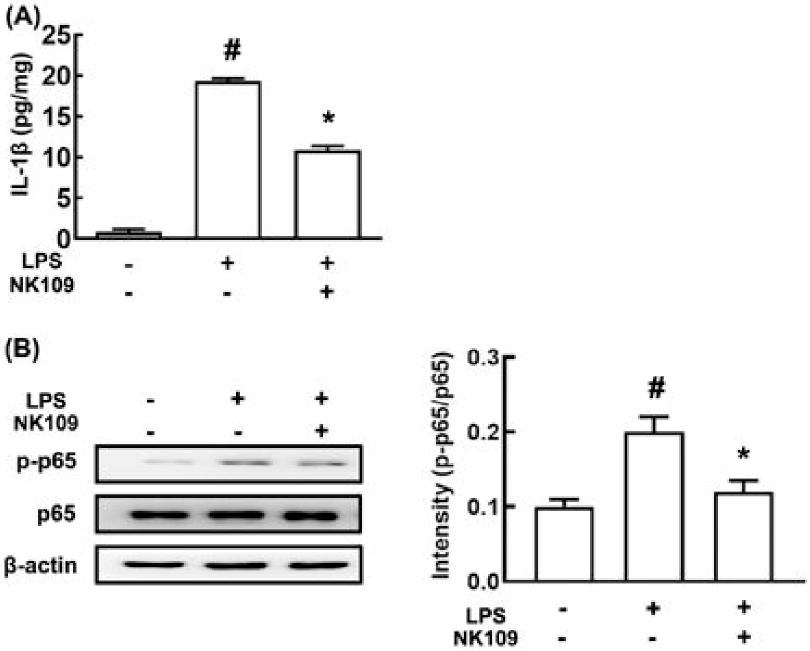

Figure S2. Effects of NK109 on the LPS-induced IL-1β and NF-κB activation in macrophages. Effect on IL-1β expression (A) and NF-κB activation (B) in macrophages. Peritoneal macrophage cells (1 × 10^6/mL) were incubated with NK109 (1×10^5 CFU/mL) in the absence or presence of LPS (100 ng/mL) for 2 h (for NK-κB) or 20 h (for IL-1β).

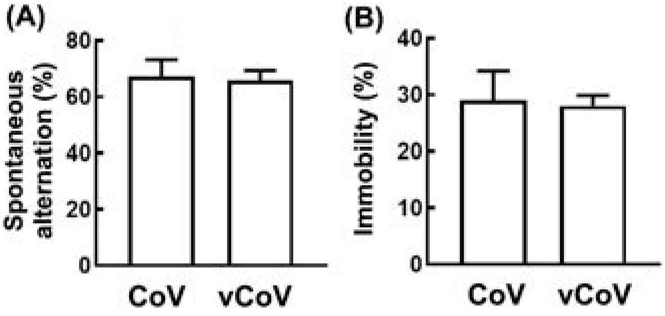

Figure S3. The difference in the cognitive impairment and depression-like behaviors between mice with and without celiac vagotomy. Cognitive impairment and depression-like behaviors were analyzed in the Y-maze task (A) and TST (B), respectively. Data values were indicated as

mean ± SD (n = 6). Means with same letters are not significantly different ($p < 0.05$).

Table S1. P-values for experimental data in the present study

Figure 1.<br>Unpaired t-test		
(A) IL-1β (pg/mL)	E.coli-/NK109- vs. E.coli+/NK109-	p=0.001
	E.coli-/NK109- vs. E.coli+/NK109+	p=0.001
	E.coli+/NK109- vs. E.coli+/NK109+	p=0.000
(B) Intensity<br>p-p65/p65	E.coli-/NK109- vs. E.coli+/NK109-	p <0.0001
	E.coli-/NK109- vs. E.coli+/NK109+	p <0.0001
	E.coli+/NK109- vs. E.coli+/NK109+	p <0.0001

Figure 2.<br>Unpaired t-test		
(A) Immobility (%)	CoV vs. EcV	p=0.001
	CoV vs ENK	p=0.331
	EcV vs ENK	p=0.003
(B) Intensity<br>p-p65/p65	CoV vs. EcV	p=0.005
	CoV vs ENK	p=0.000
	EcV vs ENK	p=0.021
(B) Intensity<br>BDNF/ β-actin	CoV vs. EcV	p=0.008
	CoV vs ENK	p=0.005
	EcV vs ENK	p=0.001
(C) IL-1β (pg/mg)	CoV vs. EcV	p=0.000
	CoV vs ENK	p=0.459
	EcV vs ENK	p=0.002
(D) cell count<br>BDNF+/NeuN+	CoV vs. EcV	p<0.0001
	CoV vs ENK	p<0.0001
	EcV vs ENK	p=0.001
(E) cell count<br>Iba1+/NKκB+	CoV vs. EcV	p<0.0001
	CoV vs ENK	p=0.868
	EcV vs ENK	p<0.0001
(F) cell count<br>IL-1R+	CoV vs. EcV	p <0.0001
	CoV vs ENK	p <0.0001
	EcV vs ENK	p <0.0001
(G) cell count<br>NKκB+/CD11c+	CoV vs. EcV	p <0.0001
	CoV vs ENK	p=0.984
	EcV vs ENK	p <0.0001
(H) MPO activity<br>(μunit/mg)	CoV vs. EcV	p=0.001
	CoV vs ENK	p=0.737

	EcV vs ENK	p=0.003
(I) IL-1β (pg/mg)	CoV vs. EcV	p=0.005
	CoV vs ENK	p=0.941
	EcV vs ENK	p=0.003
(J) Intensity p-p65/p65	CoV vs. EcV	p=0.005
	CoV vs ENK	p=0.476
	EcV vs ENK	p=0.002

Figure 3. Unpaired t-test		
(A) Spontaneous alternation (%)	CoV vs. EcV	p=0.003
	CoV vs ENK	p=0.192
	EcV vs ENK	p=0.000
(B) Immobility (%)	CoV vs. EcV	p=0.023
	CoV vs ENK	p=0.063
	EcV vs ENK	p=0.000
(C) Immobility (%)	CoV vs. EcV	p=0.001
	CoV vs ENK	p=0.026
	EcV vs ENK	p=0.025
(D) IL-1β (pg/mg)	CoV vs. EcV	p=0.393
	CoV vs ENK	p=0.016
	EcV vs ENK	p=0.028
(E) IL-6 (pg/mg)	CoV vs. EcV	p=0.370
	CoV vs ENK	p=0.001
	EcV vs ENK	p=0.030
(F) Intensity p-p65/p65	CoV vs. EcV	p <0.0001
	CoV vs ENK	p <0.0001
	EcV vs ENK	p <0.0001
(F) Intensity p-CREB/CREB	CoV vs. EcV	p=0.005
	CoV vs ENK	p=0.289
	EcV vs ENK	p=0.072
(F) Intensity BDNF/ β-actin	CoV vs. EcV	p=0.000
	CoV vs ENK	p=0.930
	EcV vs ENK	p=0.001
(G) Intensity BDNF+/NeuN+	CoV vs. EcV	p<0.0001
	CoV vs ENK	p=0.000
	EcV vs ENK	p=0.001
(H) Intensity Iba1+/NKκB+	CoV vs. EcV	P<0.0001
	CoV vs ENK	p=0.967
	EcV vs ENK	P<0.0001
(I) Intensity IL-1R+	CoV vs. EcV	p<0.0001
	CoV vs ENK	p<0.0001
	EcV vs ENK	p<0.0001
(J) CORT (mg/mL)	CoV vs. EcV	p <0.0001
	CoV vs ENK	p <0.0001
	EcV vs ENK	p <0.0001

(K) IL-6 (pg/mL)	CoV vs. EcV	p=0.006
	CoV vs ENK	p=0.006
	EcV vs ENK	p=0.074
(L) Spontaneous alternation (%)	vCoV vs. vEcV	p=0.005
	vCoV vs vENK	p=0.025
	vEcV vs vENK	p=0.193
(M) Immobility (%)	vCoV vs. vEcV	p=0.000
	vCoV vs vENK	p=0.274
	vEcV vs vENK	p=0.072
(N) Intensity p-CREB/CREB	vCoV vs. vEcV	p=0.002
	vCoV vs vENK	p=0.055
	vEcV vs vENK	p=0.045
(N) Intensity BDNF/ β-actin	vCoV vs. vEcV	p=0.039
	vCoV vs vENK	p=0.456
	vEcV vs vENK	p=0.110

Figure 4.<br>Unpaired t-test		
(A) Colon length (cm)	CoV vs. EcV	p=0.000
	CoV vs ENK	p=0.264
	EcV vs ENK	p=0.009
(B) MPO activity (μunit/mg)	CoV vs. EcV	p=0.003
	CoV vs ENK	p=0.763
	EcV vs ENK	p=0.008
(C) IL-1β (pg/mg)	CoV vs. EcV	p=0.768
	CoV vs ENK	p=0.007
	EcV vs ENK	p=0.003
(D) IL-6 (pg/mg)	CoV vs. EcV	P0.012
	CoV vs ENK	p=0.202
	EcV vs ENK	p=0.029
(E) TNF-α (pg/mg)	CoV vs. EcV	p <0.0001
	CoV vs ENK	p <0.0001
	EcV vs ENK	p <0.0001
(F) Intensity p-p65/p65	CoV vs. EcV	p=0.024
	CoV vs ENK	p=0.277
	EcV vs ENK	p=0.002
(G) cell count NKκB+/CD11c+	CoV vs. EcV	P<0.0001
	CoV vs ENK	p=0.005
	EcV vs ENK	P<0.0001
(I) OTUs	CoV vs. EcV	p=0.757
	CoV vs ENK	p=0.101
	EcV vs ENK	p=0.559
(I) Shannon	CoV vs. EcV	p=0.456
	CoV vs ENK	p=0.435
	EcV vs ENK	p=0.229

Figure 5.<br>Unpaired t-test			
(A) Spontaneous alternation (%)			
CoV vs. EcV	p=0.001	EcV vs. ENC	p=0.01
CoV vs. EtN	p=0.761	EcV vs. ENM	p=0.001
CoV vs. ENC	p=0.327	EtN vs. ENC	p=0.567
CoV vs. ENM	p=0.748	EtN vs. ENM	p=0.587
EcV vs. EtN	p=0.005	ENC vs. ENM	p=0.208
(B) Immobility (%)			
CoV vs. EcV	p <0.0001	EcV vs. ENC	p <0.0001
CoV vs. EtN	p=0.001	EcV vs. ENM	p <0.0001
CoV vs. ENC	p=0.002	EtN vs. ENC	p=0.619
CoV vs. ENM	p=0.286	EtN vs. ENM	p=0.040
EcV vs. EtN	p <0.0001	ENC vs. ENM	p=0.022
(C) Immobility (%)			
CoV vs. EcV	p <0.0001	EcV vs. ENC	p <0.0001
CoV vs. EtN	p=0.001	EcV vs. ENM	p <0.0001
CoV vs. ENC	p=0.000	EtN vs. ENC	p=0.186
CoV vs. ENM	p=0.166	EtN vs. ENM	p=0.107
EcV vs. EtN	p <0.0001	ENC vs. ENM	p=0.019
(D) IL-1β (pg/mg)			
CoV vs. EcV	p=0.000	EcV vs. ENC	p=0.047
CoV vs. EtN	p=0.006	EcV vs. ENM	p=0.002
CoV vs. ENC	p=0.000	EtN vs. ENC	p=0.102
CoV vs. ENM	p=0.018	EtN vs. ENM	p=0.044
EcV vs. EtN	p=0.006	ENC vs. ENM	p=0.005
(E) IL-6 (pg/mg)			
CoV vs. EcV	p <0.0001	EcV vs. ENC	p= p <0.0001
CoV vs. EtN	p <0.0001	EcV vs. ENM	p= p <0.0001
CoV vs. ENC	p <0.0001	EtN vs. ENC	p= p <0.0001
CoV vs. ENM	p <0.0001	EtN vs. ENM	p= p <0.0001
EcV vs. EtN	p <0.0001	ENC vs. ENM	p= p <0.0001
(F) Intensity p-p65/p65			
CoV vs. EcV	p= p <0.0001	EcV vs. ENC	p= p <0.0001
CoV vs. EtN	p=0.001	EcV vs. ENM	p= p <0.0001
CoV vs. ENC	p=0.000	EtN vs. ENC	p=0.020
CoV vs. ENM	p=0.017	EtN vs. ENM	p=0.030
EcV vs. EtN	p= p <0.0001	ENC vs. ENM	p=0.001
(F) Intensity p-CREB/CREB			
CoV vs. EcV	p=0.023	EcV vs. ENC	p=0.001
CoV vs. EtN	p=0.203	EcV vs. ENM	p=0.000
CoV vs. ENC	p=0.011	EtN vs. ENC	p=0.064
CoV vs. ENM	p=0.001	EtN vs. ENM	p=0.008
EcV vs. EtN	p=0.004	ENC vs. ENM	p=0.343
(F) Intensity BDNF/ β-actin			
CoV vs. EcV	p=0.011	EcV vs. ENC	p=0.003
CoV vs. EtN	p=0.017	EcV vs. ENM	p=0.005

CoV vs. ENC	p=0.010	EtN vs. ENC	p=0.815
CoV vs. ENM	p=0.005	EtN vs. ENM	p=0.061
EcV vs. EtN	p=0.004	ENC vs. ENM	p=0.074
(G) Intensity BDNF+/NeuN+			
CoV vs. EcV	p<0.0001	EcV vs. ENC	p<0.0001
CoV vs. EtN	p<0.0001	EcV vs. ENM	p<0.0001
CoV vs. ENC	p<0.0001	EtN vs. ENC	p=0.860
CoV vs. ENM	p<0.0001	EtN vs. ENM	p=0.004
EcV vs. EtN	p<0.0001	ENC vs. ENM	p=0.040
(H) Intensity Iba1+/NKκB+			
CoV vs. EcV	p<0.0001	EcV vs. ENC	p=0.000
CoV vs. EtN	p<0.0001	EcV vs. ENM	p<0.0001
CoV vs. ENC	p<0.0001	EtN vs. ENC	p=0.918
CoV vs. ENM	p=0.002	EtN vs. ENM	p=0.000
EcV vs. EtN	p<0.0001	ENC vs. ENM	p<0.0001
(I) Intensity IL-1R			
CoV vs. EcV	p<0.0001	EcV vs. ENC	p=0.000
CoV vs. EtN	p<0.0001	EcV vs. ENM	p<0.0001
CoV vs. ENC	p<0.0001	EtN vs. ENC	p=0.622
CoV vs. ENM	p<0.0001	EtN vs. ENM	p=0.001
EcV vs. EtN	p<0.0001	ENC vs. ENM	p<0.0001
(J) CORT (mg/mL)			
CoV vs. EcV	p <0.0001	EcV vs. ENC	p <0.0001
CoV vs. EtN	p <0.0001	EcV vs. ENM	p <0.0001
CoV vs. ENC	p <0.0001	EtN vs. ENC	p <0.0001
CoV vs. ENM	p <0.0001	EtN vs. ENM	p <0.0001
EcV vs. EtN	p <0.0001	ENC vs. ENM	p <0.0001
(K) IL-6 (pg/mL)			
CoV vs. EcV	p <0.0001	EcV vs. ENC	p <0.0001
CoV vs. EtN	p <0.0001	EcV vs. ENM	p <0.0001
CoV vs. ENC	p <0.0001	EtN vs. ENC	p <0.0001
CoV vs. ENM	p <0.0001	EtN vs. ENM	p <0.0001
EcV vs. EtN	p <0.0001	ENC vs. ENM	p <0.0001

Figure 6.<br>Unpaired t-test			
(A) Colon length (cm)			
CoV vs. EcV	p=0.001	EcV vs. ENC	p=0.135
CoV vs. EtN	p=0.021	EcV vs. ENM	p=0.003
CoV vs. ENC	p=0.003	EtN vs. ENC	p=0.049
CoV vs. ENM	p=0.087	EtN vs. ENM	p=0.152
EcV vs. EtN	p=0.010	ENC vs. ENM	p=0.009
(B) MPO activity (μunit/mg)			
CoV vs. EcV	p <0.0001	EcV vs. ENC	p=0.013
CoV vs. EtN	p <0.0001	EcV vs. ENM	p=0.015
CoV vs. ENC	p <0.0001	EtN vs. ENC	p=0.062

CoV vs. ENM	p=0.005	EtN vs. ENM	p=0.141
EcV vs. EtN	p=0.004	ENC vs. ENM	p=0.019
(C) IL-1β (pg/mg)			
CoV vs. EcV	p=0.012	EcV vs. ENC	p=0.035
CoV vs. EtN	p=0.031	EcV vs. ENM	p=0.028
CoV vs. ENC	p=0.011	EtN vs. ENC	p=0.361
CoV vs. ENM	p=0.139	EtN vs. ENM	p=0.710
EcV vs. EtN	p=0.019	ENC vs. ENM	p=0.356
(D) IL-6 (pg/mg)			
CoV vs. EcV	p=0.003	EcV vs. ENC	p=0.033
CoV vs. EtN	p=0.001	EcV vs. ENM	p=0.01
CoV vs. ENC	p=0.000	EtN vs. ENC	p=0.048
CoV vs. ENM	p=0.060	EtN vs. ENM	p=0.009
EcV vs. EtN	p=0.010	ENC vs. ENM	p=0.000
(E) TNF-α (pg/mg)			
CoV vs. EcV	p <0.0001	EcV vs. ENC	p <0.0001
CoV vs. EtN	p=0.000	EcV vs. ENM	p <0.0001
CoV vs. ENC	p=0.000	EtN vs. ENC	p=0.012
CoV vs. ENM	p=0.019	EtN vs. ENM	p=0.296
EcV vs. EtN	p <0.0001	ENC vs. ENM	p=0.006
(F) Intensity p-p65/p65			
CoV vs. EcV	p <0.0001	EcV vs. ENC	p= 0.114
CoV vs. EtN	p <0.0001	EcV vs. ENM	p= 0.000
CoV vs. ENC	p= 0.000	EtN vs. ENC	p= 0.017
CoV vs. ENM	p= 0.461	EtN vs. ENM	p= 0.002
EcV vs. EtN	p= 0.000	ENC vs. ENM	p= 0.001
(G) NKκB+/CD11c+ cell count			
CoV vs. EcV	p<0.0001	EcV vs. ENC	p<0.0001
CoV vs. EtN	p<0.0001	EcV vs. ENM	p<0.0001
CoV vs. ENC	p<0.0001	EtN vs. ENC	p=0.995
CoV vs. ENM	p=0.003	EtN vs. ENM	p=0.013
EcV vs. EtN	p<0.0001	ENC vs. ENM	p=0.033

Table S2. Effect of NK109 on the gut microbiota composition at the family level in EC- treated mice

Taxon Name	AVE			STD		
	CoV	EcV	ENK	CoV	EcV	ENK
AC160630_f	0.155	1.481	0.003*	0.242	1.686	0.005
Bacteroidaceae	23.106	10.770	31.359*	13.398	2.204	13.755
Christensenellaceae	0.688	1.434#	0.575*	0.469	0.530	0.427
Desulfovibrionaceae	0.085	1.087#	0.001*	0.133	0.517	0.004
Enterobacteriaceae	0.002	1.306#	0.006*	0.005	0.503	0.010
FR888536_f	0.066	4.523#	0.003*	0.147	4.466	0.005
Helicobacteraceae	3.428	13.638	0.046*	8.369	15.187	0.031
Lachnospiraceae	29.627	18.665	11.590	11.283	13.388	5.846
Muribaculaceae	30.006	14.061#	38.197*	12.448	7.761	15.214

Porphyromonadaceae	0.187	1.655#	0.461*	0.121	0.762	0.157
Prevotellaceae	0.129	9.461#	4.698	0.171	7.194	2.283
Rhodospirillaceae	0.011	9.763#	0.864*	0.023	10.190	0.665
Rikenellaceae	2.722	2.452	1.216	0.513	1.972	1.077
Ruminococcaceae	7.447	6.512	8.078	3.553	1.595	2.457

CoV, treated with vehicle alone; EcV, treated with K1 alone; ENK, treated with NK109 in the presence of EK. $^{\#}p<0.05$ vs. CoV group. $^{*}p<0.05$ vs. EcV group.

Table S3. Effect of NK109 on the gut microbiota composition at the genus level in EC- treated mice

Taxon Name	AVE			STD		
	CoV	EcV	ENK	CoV	EcV	ENK
Alistipes	2.707	2.396	1.211	0.529	1.963	1.075
Alloprevotella	0.000	2.540	0.000*	0.000	3.228	0.000
Bacteroides	22.249	10.770	30.140*	12.762	2.204	12.933
FR888536_g	0.066	4.523#	0.003*	0.147	4.466	0.005
Helicobacter	3.397	13.637	0.046*	8.292	15.184	0.031
KE159538_g	17.651	6.003	2.755	14.064	7.081	3.113
LARJ_g	0.010	9.721#	0.733*	0.024	10.140	0.617
Muribaculaceae_uc	1.986	0.348#	1.829*	1.144	0.402	0.582
Oscillibacter	2.063	1.207	1.582	1.457	0.607	0.872
PAC000186_g	6.900	2.431#	0.022*	3.778	1.051	0.009
PAC000198_g	1.949	0.687#	2.728*	1.082	0.351	1.688
PAC001063_g	11.953	0.080#	20.084*	8.163	0.087	14.370
PAC001066_g	2.926	1.048	8.304*	2.236	0.636	1.598
PAC001068_g	1.459	2.289	1.674	2.159	1.014	0.576
PAC001074_g	0.251	3.707#	0.020*	0.433	3.440	0.037
PAC001228_g	0.609	2.440	1.305	0.944	2.186	1.326
Paraprevotella	0.021	3.258#	0.001*	0.047	1.750	0.004
Prevotella	0.108	1.173#	4.474*	0.141	1.108	2.173
Prevotellaceae_uc	0.000	2.490	0.222	0.000	3.091	0.116
Pseudoflavonifractor	2.155	1.552	1.573	0.991	0.732	0.756

CoV, treated with vehicle alone; EcV, treated with K1 alone; ENK, treated with NK109 in the presence of EK. $^{\#}p<0.05$ vs. CoV group. $^{*}p<0.05$ vs. EcV group.

Table S4. Effect of NK109 on the gut microbiota composition at the species level in EC- treated mice.

Taxon Name	AVE			STD		
	CoV	EcV	ENK	CoV	EcV	ENK
AB599946_s	0.559	5.156#	0.024*	1.343	2.817	0.013
Bacteroides acidifaciens group	0.675	2.918	0.009*	1.180	2.185	0.013
Bacteroides caccae	0.773	0.009#	2.016*	0.764	0.009	0.659
Bacteroides_uc	19.745	0.404#	27.616*	13.955	0.434	12.244
FJ880724_s	0.021	3.207#	0.001*	0.047	1.713	0.004
Helicobacter japonicus	2.418	8.887	0.030	5.909	12.551	0.020
Helicobacter rodentium group	0.298	4.642#	0.016*	0.716	2.910	0.014

KE159538_g_uc	10.402	3.913	0.240	16.073	7.348	0.318
LARJ_g_uc	0.000	3.490	0.111	0.000	5.577	0.143
PAC000186_g_uc	2.303	0.044#	0.000*	1.564	0.023	0.000
PAC001063_g_uc	4.795	0.002#	8.532*	3.459	0.005	6.340
PAC001063_s group	7.158	0.078#	11.552*	4.728	0.086	8.041
PAC001066_g_uc	1.133	0.043#	3.767*	0.984	0.039	0.705
PAC001066_s	1.793	1.004	4.538*	1.261	0.599	0.912
PAC001074_s	0.176	2.620#	0.018*	0.312	2.571	0.030
PAC001114_s	0.008	0.128#	1.674*	0.013	0.067	0.948
PAC001120_s	5.357	1.012	0.888	8.282	2.332	1.372
PAC001267_s	0.097	0.092	1.424*	0.137	0.026	0.661
PAC001483_s	2.393	0.005#	0.001	1.875	0.002	0.004
PAC001797_s	0.000	2.584	0.002	0.000	3.787	0.004
PAC001927_s	0.000	3.528	0.000	0.000	4.773	0.000
PAC002479_s	0.000	2.501	0.000*	0.000	3.169	0.000
PAC002481_s	0.052	0.027	3.261*	0.115	0.058	1.576
PAC002482_s group	0.041	1.455	0.003*	0.101	1.693	0.005

CoV, treated with vehicle alone; EcV, treated with K1 alone; ENK, treated with NK109 in the presence of EK. #*p*<0.05 vs. CoV group. **p*<0.05 vs. EcV group.
